# DArT-based evaluation of soybean germplasm from Polish Gene Bank

**DOI:** 10.1186/s13104-021-05750-1

**Published:** 2021-08-30

**Authors:** Elzbieta Czembor, Jerzy H. Czembor, Radoslaw Suchecki, Nathan S. Watson-Haigh

**Affiliations:** 1 Plant Breeding and Acclimatization Institute–NRI, Radzikow, Blonie, Poland; 2 CSIRO Agriculture and Food, Urrbrae, SA 5064 Australia; 3 South Australian Genomics Centre, SAHMRI, North Terrace, Adelaide, SA 5000 Australia; 4 Australian Genome Research Facility, Victorian Comprehensive Cancer Centre, Melbourne, VIC 3000 Australia; 5 School of Biological Sciences, University of Adelaide, Adelaide, SA 5005 Australia; 6 Genome Informatics & Bioinformatics Training, Flagstaff Hill, SA 5159 Australia

**Keywords:** Glycine max, Genetic resources, Biodiversity, Genome-wide association study, Genomic selection, Genotyping-by-sequencing, Plant Maturity, Plant performance, Seed performance, Yield components

## Abstract

**Objective:**

Soybean is an important plant used for food, feed and many industrial purposes. Interest in soybean breeding is growing in Central Europe, including Poland. A very large number of soybean accessions are stored in gene banks, but less than 1% of them have been used for breeding. Here, we present genotypic data as well as phenotypic data on plant and seed performance, including seed chlorophyll fluorescence traits, and on yield components within a collection of soybean accessions that are conserved in the Polish Gene Bank at the Plant Breeding and Acclimatization Institute-National Research Institute.

**Results:**

The materials used consisted of sub-collections: 79 Polish genotypes, including old traditional cultivars, 24 Canadian, 21 American, 21 Swedish and 31 from Central and Eastern European Countries, 9 from France and 6 from Japan. In total, 9602 high quality SNPs were derived from DArTseq, a method utilising GBS technology. GWAS, performed with the BLINK model, revealed that a total of 41 significant SNPs were mapped for days to flowering, flower colour, plant height, days to pod formation, 100 seed weight, pod colour, seeds and hilum colour and steady-state chlorophyll fluorescence under light (Ft_Lss). This is the first report about the diversity of traditional old Polish soybean cultivars.

**Supplementary Information:**

The online version contains supplementary material available at 10.1186/s13104-021-05750-1.

## Introduction

Soybean [*Glycine max* (L.) Merr.] is one of the oldest and most important oilseed and protein crops in plant production worldwide. Soybean diversity has been extensively described in many reports, and is relatively low [1–7]. For the last decade, there has been growing interest in soybean breeding in Central Europe, including Poland. Plant breeders are constantly looking for new traits and trying to broaden the genetic basis of their materials.

Genetic resources stored in gene banks may be an important source of novel genetic variation which would be of interest to breeders if well documented and characterized with both genotyping data as well as phenotype data for traits of interest such as maturity, plant height, yield components, seed performance. To select a phenotyping method that is suitable for large-scale crop breeding research, it needs to be non-destructive and efficient; such as the steady-state chlorophyll fluorescence under light (Ft_Lss) [8].

Most studies have been conducted on Asian and American collections [2–4] with relatively few reporting on genetic diversity in European soybean collections, including Polish [5–7].

The objective of our work was to develop and implement a national management system for crop plant genetic resources as part of the AGROBANK project [9] at the Polish Gene Bank (NCPGR). It will play a leading role in incorporating phenotypic and genotypic data of crop plants of importance to Polish agriculture and food production, such as wheat, barley (http://dane.agrobank.pcss.pl/jbrowse/), soybean and pea for more effective molecular breeding of new cultivars well adapted to changing climate conditions [10, 11]. For Polish breeding companies, the presented molecular platform provides a great opportunity for more precise and efficient breeding processes to reach all major goals such as high and stable yield and resilience to all stresses caused by climate change.

## Main text

### Methods

#### Plant materials

A collection of 196 soybean accessions, including old, traditional cultivars, stored at the Polish Gene Bank (NCPGR) was evaluated using DArTseq: 80 Polish, 24 Canadian, 21 American, 21 Swedish, 31 from Central and Eastern European Countries, such as: Russia, Germany, Austria, Hungary, Ukraine and Lithuania, 9 from France, 6 from Japan, 1 from former Yugoslavia and 3 genotypes of unknown origin. The Polish accessions were selected to cover the diversity of Polish accessions held at the Gene Bank with priority given to those with key phenotypic traits in Polish breeding programs. This was then supplemented with non-Polish accessions from countries where a particular trait is most frequent. A set of 145 genotypes was preliminary evaluated under field conditions (based on the number of seeds available for each accession in the NCPGR). Passport data, listed in Additional file 1 include: accession number (ACCENUMB), accession name (ACCENAME), country of origin (ORIGCTY), institute code (INSTCODE)/institute name, acquisition date (ACQDATE), donor institution code (DONORCODE)/donor institute name (DONORNAME) and type of germplasm storage (STORAGE).

#### Plant phenotyping

Field experiment was conducted in 2019 on the experimental field of Plant Breeding and Acclimatization Institute-National Research Institute (PBAI-NRI), Radzikow near Warsaw, Poland. No specific permissions were required. No endangered or protected species were involved.

The experiment was conducted in a completely randomised design. Accessions were manually sown in 2 rows at 28 plants per row, row length of 2 m, plant spacing of 7 cm, and row spacing of 45 cm. Visual plant and seed traits were measured according to the ECPGR descriptors for soybean, with some modification (Additional file 2).

Accessions were phenotyped for: days to flowering (DAY_FLW), days to pod formation (DAY_POD_FORM), days to maturity (DTM), leaflet number (LF_NUM), leaf shape (LF_SHP), plant flower colour (FLW_CLR) and plant growth type (PL_GROW_TYPE). Days to flowering (DAY_FLW) is the number of days from planting to the day when 50% of the plants in a row have flowered, days to pod formation (DAY_POD_FORM) is the number of days from planting to the day when 50% of the plants start pod formation and days to maturity (DTM) is the number of days from planting to when 80% of pods have attained their final colour.

Thirty plants (15 per replication) were used to measure plant height (cm; PLT_HGT) after flowering time and yield components after harvesting: pods per plant (POD_PLT), seeds per pod (SED_POD), mature pod colour (POD_CLR). Hundred seed weight (g) (SED_WT) was determined in 3 replications. For visual determination of seed size and their performance, two sub-samples of 50 seeds each was used to assess seed coat colour (SED_CLR), hilum colour (HILM_CLR), seed coat surface lustro (SED_SURF_LUSTRO) and seed coat pattern (SED_PAT). To determine the steady-state fluorescence in light intensity (*Ft_Lss*), one measurement was taken using a PlantScreen™ S.C. System (Photon System Instruments—PSI) for each of 10 subsamples which themselves contained 20 seeds each.

Data reported are the mean values calculated across the 2–3 replications for a given trait. Correlation analysis was performed with the Statistica software and Excel 2019.

#### DNA extraction and quantification

1 g of young leaf tissue from the 3rd to 4th node of each seedling was excised, frozen in liquid nitrogen and stored at − 80 °C. Genomic DNA was extracted from frozen leaves using a modified cetyltrimethylammonium bromide (CTAB)/chloroform/isoamylalcohol method [12]. DNA quantification was performed by agarose gel electrophoresis (0.8%) and it was adjusted to 50 ng/μl for genotyping using DArTseq.

#### Genotyping

196 genotypes were genotyped by Diversity Arrays Technology (DArT) Pty Ltd, Australia, using DArTseq [13]. SNP calls were made against soybean [14] available in phytozome v7 and are available in Additional file 3.

#### Data filtering process

DArT data was handled using the dartR v1.1.11 package [15] in the R programming language. SNPs and genotypes were removed if SNP markers contained > 5% missing data and genotypes contained > 10% missing data, respectively. SNPs with a reproducibility score (RepAvg) < 100% were removed. Where SNPs originated from the same fragment, a random SNP was retained while the others were discarded. Non-informative monomorphic SNPs were removed, so too were rare SNPs with a minor allele frequency of < 1%.

#### Genetic diversity

Using the 9602 SNPs and the 195 genotypes passing the data filtering process, we looked at the population diversity present within the genotypes. Principle coordinate analysis (PCoA) was undertaken, using dartR, and an unrooted phylogenetic tree was constructed. The R package, ape v5.3 [16] was used to (1) calculate the pairwise distance matrix; (2) perform Neighbor-joining tree estimation; and (3) perform 100 bootstrap replicates.

#### Genome Wide Association Study (GWAS)

GWAS analysis was conducted using the GAPIT v2018.08.18 R package [17, 18] using the BLINK model [19]. We allowed GAPIT to determine the optimal number of principal components (PC) to include as covariates for each of 24 phenotypes. Physical genome positions of markers were derived from the DArTseq SNP genotype file. Since GAPIT can only handle complete data, only markers with a physical position on one of the 20 chromosomes and zero missing data were used as input to the GWAS analysis.

### Results

#### Phenotypic traits

This is the first report in which detailed phenotypic traits have been detailed in Polish soybean accessions, taking into account plant morphology, yield components and many seed performance traits. Commercially important appearance quality traits include mature pod colour, seed coat colour, hilum colour, seed coat surface, seed coat pattern and have been evaluated for all genotypes.

Flowering time, time to pod formation and maturity are important for improving the adaptability and yield of seed crops in different environments. The variation difference for these traits were 7.53%, 8.54% and 7.11% respectively. A wide variation was detected for yield components: pods per plant (POD_PLT) with a coefficient of variation (CV) of 46.7% (range 16.0–91.7) and for 100-seed with CV 16.1% (range 3.2–29.0 g).

All seed performance traits showed remarkable variation: seed coat colour—66.24% (from black or brown to green or yellow), seed coat surface lustro—24.8% (from shiny to heavy bloom), seed coat pattern–212.4% (from light hilum to striped), hilum colour—49.3% (from and seed thickness—17.2%). The black seeds are uncommon and found in only a few genotypes originating from Poland and the USA. Raw phenotypic data and summary points of trait values are available in Additional files 2 and 4 respectively. Hundred seed weight was negatively correlated with flowering time, plant height, pods per plants and seed coat colour (− 0.541; p ≤ 0.05) (Additional file 5). *Ft_Lss* measurements by PlantScreen™ S.C. System was significantly correlated with visual assessments (− 0.732, p ≤ 0.05) but with more rapid and higher throughput of measurements. Suggesting it to be a fast and accurate method by which seed coat colour and uniformity can be characterised for many seeds samples stored at the Gene Bank. *Ft_Lss* is moderately positively correlated with 100-seed weight (0.420, p ≤ 0.050). This information can form the basis on which to drive genetic improvement in soybean.

#### Genetic diversity

A total of 20,600 SNP markers across 196 genotypes were generated by DArTseq. Of these, 2613 (12.7%) markers had no missing data while 16,055 (77.9%) of markers had missing calls in less than 10 genotypes. One genotype was removed (199S) due to having more than 10% missing calls among the 16,055 markers. A further 3370 monomorphic markers were removed together with 2655 markers which were not 100% reproducible according to the DArT metric RepAvg. Where a DArTseq tag contained more than one SNP, one was selected at random. In total 9602 SNP markers and 195 genotypes were retained for further analysis.

**Fig. 1:**
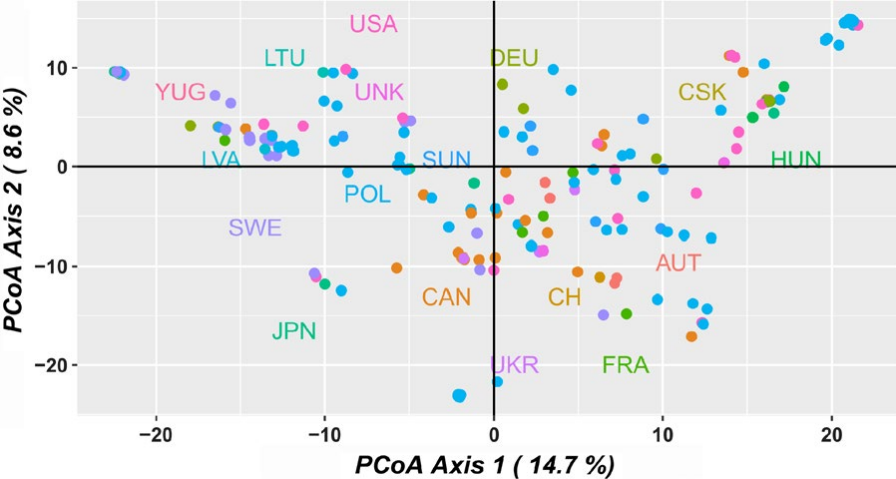
Diversity of the 195 genotypes coloured by country of origin

The PCoA (Fig. 1) and Neighbour Joining tree (Additional file 6) shows a set of genotypes with minimal clustering around the country of origin while the Polish accessions capture much of the diversity observed across this set of genotypes. The analysed Polish accessions represent a diverse and previously uncharacterised source of genetic material for use in soybean breeding programs.

#### Marker-trait associations using BLINK

Of the 9602 SNPs, 6000 markers had complete data (position and genotype calls) and were evenly spread across the 20 chromosomes with between 220 (chr11) and 391 (chr8) markers per chromosome.

**Fig. 2:**
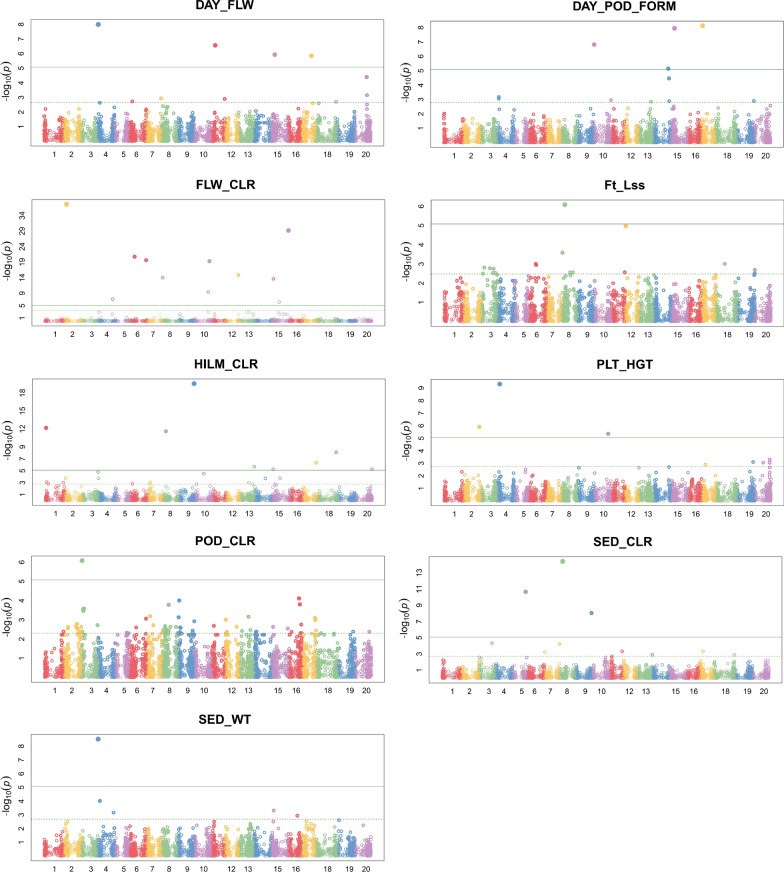
Manhattan plots for each phenotypic trait which showed a significant marker-trait association

Of the 24 phenotypes analysed, 9 were found to have at least 1 marker-trait associations with a total of 41 markers associated with those traits (Fig. 2). Additional file 7 contains details of the markers associated with the traits, together with physical position, allele information and sequences flanking the markers.

### Discussion

Soybean is one of the oldest and most important oilseed and protein crops worldwide. It was domesticated in China and is now grown on all other continents. In the last decade, there is growing interest in growing of soybean in Central Europe including Poland. However, most of the varieties/lines previously cultivated in Poland were imported from Canada. These are not well adapted to the prevailing continental climate and has led to disappointing yields and late maturity. Appearance quality traits play an important role in the production and export of soybean as well as impacting on their commercial value.

Many breeding programs are looking for new sources of variation for specific traits, especially to make genetic progress in adaptation to rapidly changing environmental conditions. Although soybean diversity among Asian and American collections has been extensively studied, diversity is low [1–4]. While very little data on the genetic diversity of European soybean collections has been published [5–7]. Tavaud-Pirra et al*.* [20] screened the diversity of 350 cultivated soybean genotypes originating from various European countries. These included 185 accessions from the INRA soybean collection, and 32 cultivars and recent breeding lines used in Western Europe from 1950 to 2000. The results indicated relatively low genetic diversity, albeit based on a relatively small number of markers, among West European breeding lines. As such, there is growing interest in providing breeders with detailed information on which to select accessions for bringing in novel sources of genetic variation for important commercial traits. Here we provided genotypic and phenotypic information on a diverse set of previously uncharacterised Polish Gene Bank accessions which compliments information from other global soybean collections.

## Limitations

The number of seeds available for each accession in the NCPGR was limited. As such it was only possible to conduct an experiment in one environment. Replication of the experiment and phenotyping of traits in multi-environments is advisable.

## Supplementary Information

**Additional file 1.** Passport data.

**Additional file 2.** Phenotype data.

**Additional file 3.** Genotype (DArTseq) data.

**Additional file 4.** Phenotype summary plots for traits with marker-trait associations (MTAs).

**Additional file 5.** Pearson correlation coefficients of phenotypic traits.

**Additional file 6.** Unrooted NJ tree with 100 bootstrap replicates. Only bootstrap values ≥ 80% are shown.

**Additional file 7.** Table of significant marker-trait associations (MTAs).

## Data Availability

All data generated or analysed during this study are included in this published article and its Additional files.

## Abbreviations

ACCENAME: Accession name
ACCENUMB: Accession number
ACQDATE: Acquisition date
BLINK: Bayesian-information and Linkage-disequilibrium Iteratively Nested Keyway
DAY_FLW: Days to flowering
DAY_POD_FORM: Days to pod formation
DONORCODE: Donor institution code
DONORNAME: Donor institute name
DTM: Days to maturity
FLW_CLR: Plant flower colour
Ft_Lss: Steady-state chlorophyll fluorescence under light
GBS: Genotyping-By-Sequencing
HILM_CLR: Hilum colour
INSTCODE: Institute code/institute name
LF_NUM: Leaflet number
LF_SHP: Leaf shape
MTA: Marker-trait association
NCPGR: National Centre for Plant Genetic Resources
ORIGCTY: Country of origin
PBAI-NRI: Plant Breeding and Acclimatization Institute-National Research Institute
PCoA: Principle coordinate analysis
PL_GROW_TYPE: Plant growth type
PLT_HGT: Plant height (cm)
POD_CLR: Mature pod colour
POD_PLT: Pods per plant
SED_CLR: Seed coat colour
SED_PAT: Seed coat pattern
SED_POD: Seeds per pod
SED_SURF_LUSTRO: Seed coat surface lustro
SED_WT: 100 Seed weight (g)
STORAGE: Type of germplasm storage
