# Supplementary material for: DArT-based evaluation of soybean germplasm from Polish Gene Bank
Source: BMC Res Notes. 2021 Aug 30;14:343. doi: 10.1186/s13104-021-05750-1 (PMC8404325; doi:10.1186/s13104-021-05750-1)
Supplement: Supplementary file 4 — Additional file 4. Phenotype summary plots for traits with marker-trait associations (MTAs). [file 13104_2021_5750_MOESM4_ESM.docx]

Additional file 4

**Days to Flowering (DAY_FLW)**

**Cumulative Distribution of DAY_FLW**

**Genotype Index**

**Days to Flowering**

**Days to Flowering**

**Days to Flowering**

**Days to Flowering**

**Boxplot of DAY_FLW**

**Histogram of DAY_FLW**

**DAY_FLW by Genotype Index**

Phenotype summary plots for DAY_FLW.

**Cumulative Distribution of DAY_POD_FORM**

**DAY_POD_FORM by Genotype Index**

**Genotype Index**

**Days to Pod Formation (DAY_POD_FORM)**

**Days to Pod Formation**

**Days to Pod Formation**

**Days to Pod Formation**

**Days to Pod Formation**

**Boxplot of DAY_POD_FORM**

**Histogram of DAY_POD_FORM**

Phenotype summary plots for DAY_POD_FORM.

**Cumulative Distribution of FLW_CLR**

**Genotype Index**

**Flower Colour**

**Flower Colour (FLW_CLR)**

**FLW_CLR by Genotype Index**

**Histogram of FLW_CLR**

**Boxplot of FLW_CLR**

**Flower Colour**

**Flower Colour**

**Flower Colour**

Phenotype summary plots for FLW_CLR.

**Cumulative Distribution of Ft_Lss**

**Genotype Index**

**Ft_Lss**

**Steady-state fluorescence in light intensity (****Ft_Lss)**

**Ft_Lss by Genotype Index**

**Histogram of FT_Lss**

**Boxplot of Ft_Lss**

**Ft_Lss**

**Ft_Lss**

**Ft_Lss**

Phenotype summary plots for Ft_Lss.

**Cumulative Distribution of HILM_CLR**

**Genotype Index**

**Hilum Colour (HILM_CLR)**

**HILM_CLR by Genotype Index**

**Histogram of HILM_CLR**

**Boxplot of HILM_CLR**

**Hilum Colour**

**Hilum Colour**

**Hilum Colour**

**Hilum Colour**

Phenotype summary plots for HILM_CLR.

**Cumulative Distribution of PLT_HGT**

**Genotype Index**

**Plant Height (cm)**

**Plant Height (PLT_HGT)**

**Plant Height (cm)**

**Plant Height (cm)**

**Plant Height (cm)**

**Boxplot of PLT_HGT**

**Histogram of PLT_HGT**

**PLT_HGT by Genotype Index**

Phenotype summary plots for PLT_HGT.

**Cumulative Distribution of POD_CLR**

**Genotype Index**

**Pod Colour (POD_CLR)**

**POD_CLR by Genotype Index**

**Histogram of POD_CLR**

**Boxplot of POD_CLR**

**Pod Colour**

**Pod Colour**

**Pod Colour**

**Pod Colour**

Phenotype summary plots for POD_CLR.

**Cumulative Distribution of SED_CLR**

**Genotype Index**

**SED_CLR by Genotype Index**

**Histogram of SED_CLR**

**Boxplot of SED_CLR**

**Seed Colour**

**Seed Colour**

**Seed Colour**

**Seed Colour (SED_CLR)**

**Seed Colour**

Phenotype summary plots for SED_CLR.

**Cumulative Distribution of SED_WT**

**Genotype Index**

**100-Seed Weight (SED_WT)**

**SED_WT by Genotype Index**

**Histogram of SED_WT**

**Boxplot of SED_WT**

**100-Seed Weight (g)**

**100-Seed Weight (g)**

**100-Seed Weight (g)**

**100-Seed Weight (g)**

Phenotype summary plots for SED_WT.
