# Supplementary material for: DArT-based evaluation of soybean germplasm from Polish Gene Bank
Source: BMC Res Notes. 2021 Aug 30;14:343. doi: 10.1186/s13104-021-05750-1 (PMC8404325; doi:10.1186/s13104-021-05750-1)
Supplement: Supplementary file 6 — Additional file 6. Unrooted NJ tree with 100 bootstrap replicates. Only bootstrap values ≥ 80% are shown. [file 13104_2021_5750_MOESM6_ESM.pdf]

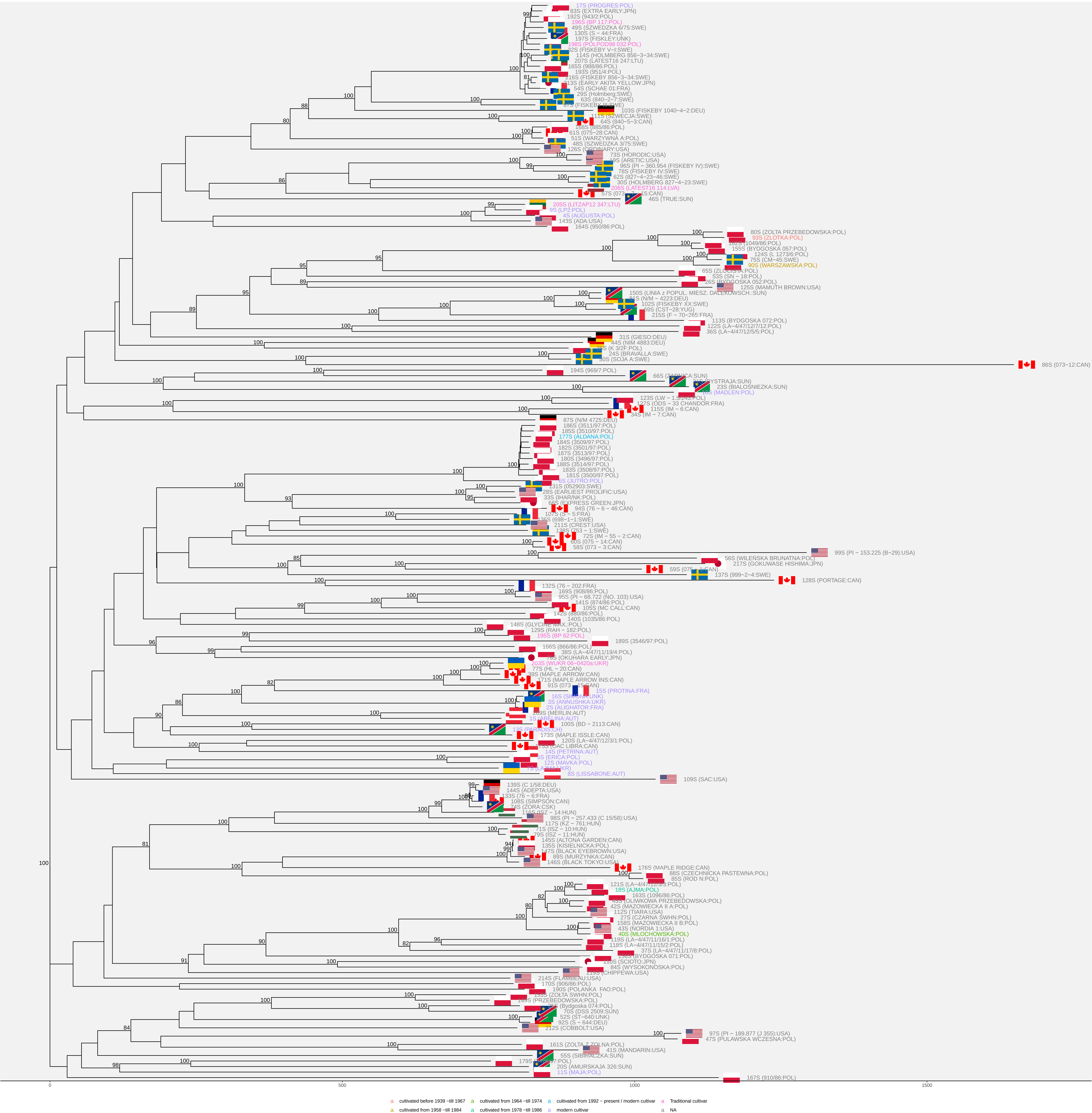
17S (PROGRES:POL)
83S (EXTRA EARLY:JPN)
192S (943/2:POL)
196S (BP 117:POL)
49S (SZWEDZKA 6/75:SWE)
130S (S − 44:FRA)
197S (FISKLEY:UNK)
198S (POLPOD98 032:POL)
32S (FISKEBY V−I:SWE)
114S (HOLMBERG 856−3−34:SWE)
207S (LATEST16 247:LTU)
165S (988/86:POL)
193S (951/4:POL)
216S (FISKEBY 856−3−34:SWE)
213S (EARLY AKITA YELLOW:JPN)
54S (SCHAE 01:FRA)
29S (Holmberg:SWE)
63S (840−2−7:SWE)
103S (FISKEBY 1040−4−2:DEU)
111S (SZWECJA:SWE)
64S (840−5−3:CAN)
168S (885/86:POL)
61S (075−28:CAN)
51S (WARZYWNA A:POL)
48S (SZWEDZKA 3/75:SWE)
73S (HORODIC:USA)
96S (PI − 360.954 (FISKEBY IV):SWE)
78S (FISKEBY IV:SWE)
62S (827−4−23−46:SWE)
30S (HOLMBERG 827−4−23:SWE)
206S (LATEST16 114:LVA)
46S (TRUE:SUN)
205S (LITZAP12 347:LTU)
9S (LP2:POL)
4S (AUGUSTA:POL)
143S (ADA:USA)
164S (950/86:POL)
80S (ZOLTA PRZEBEDOWSKA:POL)
93S (ZLOTKA:POL)
155S (BYDGOSKA 057:POL)
124S (L 1273/6:POL)
75S (CM−45:SWE)
90S (WARSZAWSKA:POL)
53S (SN − 18:POL)
125S (MAMUTH BROWN:USA)
102S (FISKEBY XX:SWE)
69S (CST−28:YUG)
215S (F − 70−265:FRA)
113S (BYDGOSKA 072:POL)
122S (LA−4/47/12/7/12:POL)
36S (LA−4/47/12/5/5:POL)
31S (GIESO:DEU)
44S (NIM 4883:DEU)
24S (BRAVALLA:SWE)
86S (073−12:CAN)
194S (969/7:POL)
23S (BIALOSNIEZKA:SUN)
127S (ODS − 33 CHANDOR:FRA)
115S (IM − 6:CAN)
186S (3511/97:POL)
185S (3510/97:POL)
177S (ALDANA:POL)
184S (3509/97:POL)
182S (3501/97:POL)
187S (3513/97:POL)
180S (3496/97:POL)
188S (3514/97:POL)
183S (3508/97:POL)
181S (3500/97:POL)
131S (052903:SWE)
28S (EARLIEST PROLIFIC:USA)
33S (IHAR/NK:POL)
68S (EXPRESS GREEN:JPN)
94S (76 − 6 − 46:CAN)
107S (S − 5:FRA)
136S (698−1−1:SWE)
211S (CREST:USA)
138S (753 − 1:SWE)
72S (IM − 55 − 2:CAN)
60S (075 − 14:CAN)
58S (073 − 3:CAN)
99S (PI − 153.225 (B−29):USA)
56S (WILENSKA BRUNATNA:POL)
217S (GOKUWASE HISHIMA:JPN)
137S (999−2−4:SWE)
128S (PORTAGE:CAN)
132S (76 − 202:FRA)
169S (908/86:POL)
95S (PI − 68.722 (NO. 103):USA)
141S (874/86:POL)
105S (MC CALL:CAN)
140S (1035/86:POL)
129S (RAH − 182:POL)
195S (BP 62:POL)
189S (3546/97:POL)
166S (866/86:POL)
38S (LA−4/47/11/19/4:POL)
76S (OKUHARA EARLY:JPN)
203S (WUKR 06−0420a:UKR)
77S (HL − 20:CAN)
39S (MAPLE ARROW:CAN)
171S (MAPLE ARROW INS:CAN)
15S (PROTINA:FRA)
3S (ANNUSHKA:UKR)
2S (ALIGHATOR:FRA)
209S (MERLIN:AUT)
1S (ABELINA:AUT)
100S (BD − 2113:CAN)
173S (MAPLE ISSLE:CAN)
120S (LA−4/47/12/3/1:POL)
14S (PETRINA:AUT)
12S (MAVKA:POL)
8S (LISSABONE:AUT)
109S (SAC:USA)
139S (C 1/58:DEU)
144S (ADEPTA:USA)
133S (76 − 6:FRA)
108S (SIMPSON:CAN)
74S (ZORA:CSK)
116S (ISZ − 14:HUN)
98S (PI − 257.433 (C 15/58):USA)
117S (KZ − 761:HUN)
71S (ISZ − 10:HUN)
79S (ISZ − 11:HUN)
145S (ALTONA GARDEN:CAN)
135S (KISIELNICKA:POL)
147S (BLACK EYEBROWN:USA)
89S (MURZYNKA:CAN)
146S (BLACK TOKYO:USA)
176S (MAPLE RIDGE:CAN)
88S (CZECHNICKA PASTEWNA:POL)
85S (ROD N:POL)
18S (AJMA:POL)
163S (1096/86:POL)
42S (MAZOWIECKA II A:POL)
112S (TIARA:USA)
27S (CZARNA SWHN:POL)
158S (MAZOWIECKA II B:POL)
43S (NORDIA 1:USA)
40S (MLOCHOWSKA:POL)
119S (LA−4/47/11/16/1:POL)
118S (LA−4/47/11/15/2:POL)
37S (LA−4/47/11/17/8:POL)
110S (SCIOTO:JPN)
84S (WYSOKONOSKA:POL)
214S (FLAMBEAU:USA)
170S (906/86:POL)
190S (POLANKA FAO:POL)
70S (DSS 2509:SUN)
52S (ST−640:UNK)
92S (S − 644:DEU)
212S (COBBOLT:USA)
97S (PI − 189.877 (J 355):USA)
47S (PULAWSKA WCZESNA:POL)
41S (MANDARIN:USA)
20S (AMURSKAJA 326:SUN)
11S (MAJA:POL)
167S (910/86:POL)
0
500
1000
1500
cultivated before 1939 −till 1967
cultivated from 1958 −till 1984
cultivated from 1964 −till 1974
cultivated from 1978 −till 1986
cultivated from 1992 − present / modern cultivar
modern cultivar
Traditional cultivar
NA
